# Design and Preparation of Imidazole Ionic Liquid-Based Magnetic Polymers and Its Adsorption on Sunset Yellow Dye

**DOI:** 10.3390/ma15072628

**Published:** 2022-04-02

**Authors:** Yafei Liu, Yiyu Shi, Yan Cui, Fen Zhao, Mindong Chen

**Affiliations:** Collaborative Innovation Center of Atmospheric Environment and Equipment Technology, Jiangsu Key Laboratory of Atmospheric Environment Monitoring and Pollution Control, School of Environmental Science and Engineering, Nanjing University of Information Science & Technology, Nanjing 210044, China; lllyf9810@163.com (Y.L.); syyee@163.com (Y.S.); cuiyan@nuist.edu.cn (Y.C.); zhaofen1001@163.com (F.Z.)

**Keywords:** magnetic polymer, ionic liquids, adsorption, reusability

## Abstract

Magnetic polymers are often used as loading materials for ionic liquids because of their excellent magnetic separation properties. In this study, a novel imidazolium-based ionic liquid-modified magnetic polymer was synthesized by suspension polymerization and grafting, denoted as γ-Fe_2_O_3_@GMA@IM, and this magnetic polymer was used for the adsorption of the acid dye FCF. The magnetic polymer was characterized by SEM, FTIR, XRD, VSM and TGA. These techniques were used to reveal the overall physical properties of magnetic polymers, including the presence of morphology, functional groups, crystalline properties, magnetism and thermal stability. Studies have shown that γ-Fe_2_O_3_@GMA@IM can adsorb FCF in a wide pH range (2–10), with a maximum adsorption capacity of 445 mg/g. The adsorption data were more in line with the pseudo-second-order kinetic model and the Freundlich isotherm. In order to investigate its reusability, this study used 10% NaCl as the desorption solution, and carried out five batches of adsorption–desorption cycles. After five cycles, the adsorption effect was maintained at 98.3%, which showed a good recycling performance.

## 1. Introduction

Ionic liquid is an ionic compound composed of organic cations and organic or inorganic anions, caused by the asymmetry of the volume and structure of anions and cations, and their delocalized positive charges reduce the interactions between anions and cations, making packing crystallization difficult; ionic liquids have a low melting point and present a liquid state at or near room temperature. Ionic liquids have unique physical and chemical properties, such as non-volatile properties, high electrical conductivity, excellent solubility and good stability; therefore, they are a popular topic in green chemistry research and have been widely studied in catalysis [1], adsorption [2], extraction and separation [3,4], and electrochemistry [5,6].

However, ionic liquids have some application issues, such as easy loss and difficult separation, which greatly limits their use. In order to solve these problems, many researchers have carried out research on loaded ionic liquids. A loaded ionic liquid refers to a solid substance with an ionic liquid structure obtained by physically or chemically loading the ionic liquid on organic or inorganic materials, such as polymers [7], magnetic materials [8], and silica gel [9]. Among them, magnetic materials have attracted extensive attention due to their excellent properties, such as large specific surface area, high surface reactivity, high catalytic efficiency, strong adsorption performance, and magnetic response.

Mu et al. [8]. prepared novel magnetic ionic liquid-based nanocomposites [NiFe_2_O_4_@BMSI]Br and [NiFe_2_O_4_@BMSI]HSO_4_ with 1-butylimidazole, (3-bromopropyl)-trimethoxy silane and NiFe_2_O_4_ nanoparticles for use in catalytic vegetable oil exchange to prepare biodiesel, which showed a good catalytic activity and regenerative utilization. Sara et al. [10]. determined lead in drinking water by FI-ICP-OES after the preconcentration of ionic liquid-modified magnetic nanoparticles. Tang et al. [11]. synthesized multifunctional magnetic chitosan-graphene oxide-ionic liquid ternary nanohybrids with Fe_3_O_4_, chitosan and 1-octyl-3-vinylimidazolium bromide for the efficient adsorption of alkaloids. Jiang et al. [12]. synthesized Fe_3_O_4_@SiO_2_@ILs with Fe_3_O_4_, tetraethyl silicate and 1-butyl-3-methylimidazolium monosodium phosphate, which could rapidly extract Hg^2+^ from natural water with the aid of ultrasound. Latifeh et al. [13] modified the SiO_2_-coated magnetic core with 3-chloropropyltrimethoxysilane, and then modified it with N-methylimidazole-hexafluorophosphate. The synthesized material was used to adsorb paraquat in water, showing a good dispersion stability and adsorption performance.

Sunset yellow (FCF) is one of most common used dyes in varies of the industry. It is a monoazide disulfonated hydroxyl dye extracted from petroleum and mainly used in food production for juice, soft drinks, candy, jellies and salty snacks, giving it a yellow–orange color [14]. Studies have shown that FCF may have the following adverse effects: allergies, attention deficit, and hyperactivity [15]. Understanding how to remove FCF from water is extremely necessary. The adsorption method is regarded as one of the most promising and effective methods because of its simple operation, low cost and no other harmful substances. Many adsorbent materials have been applied to the removal of FCF, such as mesoporous molecular sieves, commercial activated carbon [16,17], chitosan, graphene [18], or more complex nanocomposites, such as magnetic nanoparticles, modified halloysite nanotubes/poly([2-(propylene) acyloxy)ethyl]trimethylammonium chloride) hybrid particles [19], magnetically functionalized graphene oxide, and cetyltrimethylammonium bromide nanocomposites, etc. [20]. However, most of these studies show a low adsorption capacity and unsatisfactory regeneration performance, which needs to be improved.

In this study, the silane coupling agents, TEOS and DMDES, were used to coat γ-Fe_2_O_3_ twice to improve its corrosion resistance in acidic water. Magnetic polymer γ-Fe_2_O_3_@GMA was obtained by suspension polymerization with glycidyl methacrylate (GMA) as monomer and divinylbenzene (DVB) as crosslinking agent. Finally, γ-Fe_2_O_3_@GMA@IM was obtained by loading imidazolium ionic liquid on the magnetic polymer via the grafting method. The anionic dye FCF is adsorbed and removed by the magnetic polymer. The physicochemical properties of the polymer and the effect of the initial pH on the adsorption performance were investigated. The adsorption behavior and mechanism were investigated by adsorption kinetics and adsorption isotherms, and by examining its reproducible adsorption–desorption process over multiple cycles capital to determine its reproducibility.

## 2. Materials and Method

### 2.1. Materials and Characteristics

In terms of materials, γ-Fe_2_O_3_ magnetic powder, tetraethyl silicate (TEOS, 98%), divinylbenzene (DVB, 66.3%), isopropyl tris(dioctylphosphoryloxy) titanate (98%), gelatin (photographic grade), and ammonia water were purchased from Shanghai Aladdin Biochemical Technology Co., Ltd. (Shanghai, China); glycidyl methacrylate (GMA, 99%), cyclohexanol (99%), polyvinyl alcohol (type 1788), and 1-methylimidazole hydrochloride (97%) were purchased from Beijing Huawei Ruike Chemical Co., Ltd. (Beijing, China); benzoyl peroxide (BPO, 99%) and sunset yellow (FCF, 87%) were provided by Shanghai McLean Biotechnology Co., Ltd. (Shanghai, China); dimethyldiethoxysilane (DMDES, 98%) was purchased from Shanghai Merrill Chemical Technology Co., Ltd. (Shanghai, China); and sodium chloride was obtained from Nanjing Haitai Scientific Equipment Co., Ltd. (Nanjing, China).

The functional groups of the obtained magnetic polymers were characterized by Fourier transform infrared spectroscopy (FTIR) (Thermo Fisher, Waltham, MA, USA). The size and morphology of the polymers were determined by scanning electron microscopy (SEM) (HITACHI, Tokyo, Japan). The crystalline and amorphous properties of the polymers were determined using X-ray diffraction techniques (XRD) (Shimazu, Kyoto, Japan). Physicochemical properties, such as specific surface area and pore size, were measured by Brunner−Emmet−Teller (BET) (*Quantachrome,* Boynton Beach, FL, USA). The magnetic properties of magnetic polymers were determined by a vibrating sample magnetometer (VSM) (Lake Shore, Columbus, OH, USA). Thermogravimetric analyzer (TGA) (Thermo Fisher, Waltham, MA, USA) was used to determine the thermal stability and melting/decomposition temperature of the polymers from room temperature to 600 °C under N_2_ atmosphere. The charge properties of polymers at different pH were determined by Zeta potential (MALVERN, Birmingham, UK).

### 2.2. Preparation of Magnetic Polymers

**Figure 1:**
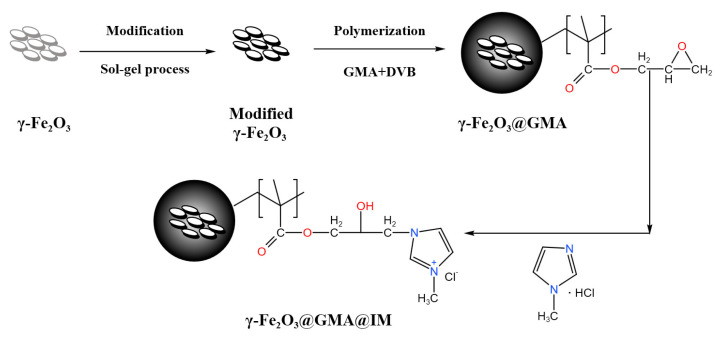
Preparation roadmap of magnetic polymer γ-Fe_2_O_3_@GMA@IM.

The preparation route of the ionic liquid-modified magnetic polymer γ-Fe_2_O_3_@GMA@IM is shown in Figure 1. There are 3 steps in this process, including sol–gel modification, suspension polymerization and the grafting reaction of magnetic powder.

#### 2.2.1. Surface Modification of γ-Fe_2_O_3_

Next, 40.0 g γ-Fe_2_O_3_ was added to a conical flask containing 150 mL ethanol following these steps: add 1 mL of ammonia water, stir at 40 °C for 10 min, add 2 mL of TEOS, stir for 30 min, add 2 mL of TEOS and 2 mL of DMDES, and stir for 2 h. After filtration, the solution was washed with ethanol many times and vacuum-dried at 60 °C for 12 h to obtain about 40.527 g of modified γ-Fe_2_O_3_.

#### 2.2.2. Preparation of γ-Fe_2_O_3_@GMA

In a 250 mL flask, 20 g GMA and 4 g DVB were added and stirred evenly. Then, 0.4 g BPO, 0.5 g isopropyl tris (dioctyl phosphate acyloxy) titanate, 8 g γ-Fe_2_O_3_, and 20 g cyclohexanol were added and stirred at 40 °C for 30 min. Lastly, 120 mL of the water phase (1 g gelatin, 14 g sodium chloride, 0.1 g polyvinyl alcohol) was added and heated to 80 °C. After 8 h, the solution was washed with hot water and dried to obtain about 25.179 g γ-Fe_2_O_3_@GMA.

#### 2.2.3. Preparation of γ-Fe_2_O_3_@GMA@IM

Subsequently, 5 g γ-Fe_2_O_3_@GMA was added to 100 mL of 10% 1-methylimidazole hydrochloride solution, stirred at pH 8–10 at 70 °C for 10 h, washed with clean water, extracted with methanol and acetone for Soxhlet for 12 h, soaked in 15% sodium chloride solution for 2 h, washed with distilled water until there was no chlorine ion in the effluent, and the powders were placed under vacuum at 50 °C to obtain about 6.931 g γ-Fe_2_O_3_@GMA@IM.

### 2.3. Batch Adsorption Studies

In this study, UV-Vis spectrophotometry was used to measure the concentration of FCF solution, and the measurement wavelength of FCF was 482 nm. Meanwhile, in order to ensure the accuracy and reproducibility of the experiment, all adsorption experiments were repeated three times to calculate the average value under the guarantee of the adsorption data error of ±3%.

#### 2.3.1. Contrast Experiment

In order to investigate the effect of intermediate products on FCF adsorption, 0.100 g modified γ-Fe_2_O_3_, γ-Fe_2_O_3_@GMA, and γ-Fe_2_O_3_@GMA @IM were added to a conical flask containing 100 mL FCF solution with a concentration of 100 mg/L, respectively. The conical flask was shaken at 25 °C for 24 h at 150 rpm, and the equilibrium adsorption quantity Q_e_ (mg/g) was calculated according to Formula (1):

Q_e_ = V·(C_0_ − C_e_)/W (1)

where V (L) is the solution volume, W (g) is the mass of the adsorbent, and C_0_ (mg/g) and C_e_ (mg/g) are the initial dye concentration and the equilibrium concentration of adsorption, respectively.

#### 2.3.2. Effect of pH

Then, 100 mL of FCF solution with concentrations of 200 and 300 mg/L was added to the erlenmeyer flask, followed by 0.100 g γ-Fe_2_O_3_@GMA@IM. The pH was adjusted to 2–10 with HCl and NaOH. The conical flask was shaken at 25 °C for 24 h to calculate the equilibrium adsorption quantity Q_e_ (mg/g) according to Formula (1).

#### 2.3.3. Adsorption Kinetics

For kinetic studies, 300 mL of FCF at a concentration of 200 mg/L and 0.300 g of γ-Fe_2_O_3_@GMA@IM were placed in a conical flask and stirred in a shaker flask at 25 °C, 150 rpm. At the designed time interval, 1 mL of solution was taken and the residual dye concentration was measured with a UV-Vis spectrophotometer (MAPADA, Shanghai, China). The experiment was repeated three times. The adsorption amount Q_t_ (mg/g) was calculated according to Formula (1).

#### 2.3.4. Adsorption Isotherm

A measure of 0.100 g of γ-Fe_2_O_3_@GMA@IM was poured into FCF solutions at different concentrations and shaken at 150 rpm for 24 h at 25 °C, 35 °C, and 45 °C, respectively. According to the equilibrium concentration of the solution, the adsorption isotherm data at the temperature could be obtained. When the adsorption was in equilibrium, the equilibrium adsorption amount was calculated by Formula (1).

#### 2.3.5. Regeneration Experiment

Next, 50 mL of FCF solution with a concentration of 200 mg/L was added to the conical flask, then 0.050 g γ-Fe_2_O_3_@GMA@IM was added. The flask was shaken at 25 °C for 24 h, separated by a magnet, and ultraviolet-visible spectrophotometry was used to measure the concentration of the solution after the reaction. Then, 10% NaCl was added and shaken at 298 K for 24 h to complete desorption. Five cycles of the adsorption–desorption process were completed to evaluate the reusability of magnetic polymers. The regeneration efficiency was calculated by Formula (2):

R_E_ = Q_i_/Q_0_·100% (2)

where Q_i_ and Q_0_ (mg/g) are the adsorption capacities of the regenerated adsorbent and the original adsorbent, respectively.

## 3. Results and Discussion

### 3.1. Description

The physicochemical properties of the ionic liquid-modified magnetic polymers are shown in Table 1. It can be seen that the substance has a large specific surface area, which can effectively increase the contact area between the dye and the polymer, improving the absorption effect of the dye.

**Figure 2:**
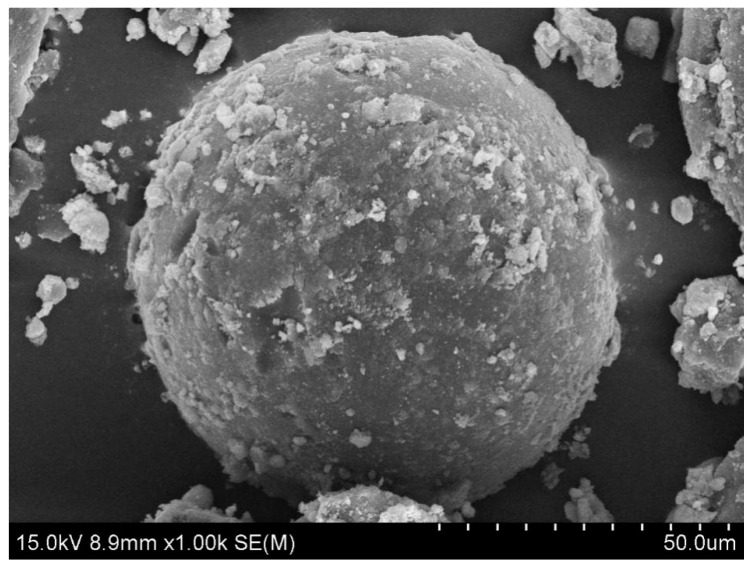
SEM image of γ-Fe_2_O_3_@GMA@IM.

The surface morphology of γ-Fe_2_O_3_@GMA@IM is shown in Figure 2. Due to the existence of the inorganic magnetic powder γ-Fe_2_O_3_, the surface of the magnetic polymer is relatively rough, with many irregular protrusions and some concave holes on the surface of the polymer, corresponding to its larger specific surface area.

**Figure 3:**
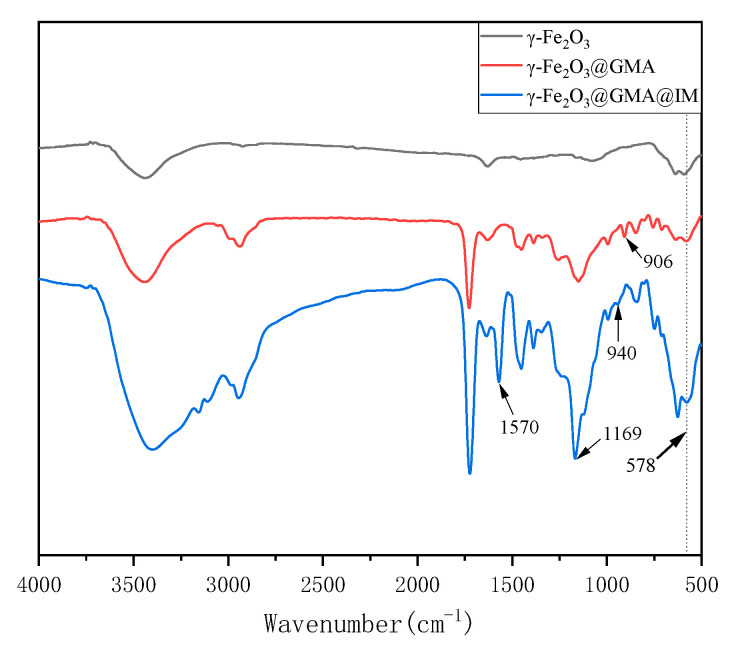
FTIR diagram of γ-Fe_2_O_3_@GMA@IM.

The FTIR of γ-Fe_2_O_3_@GMA and γ-Fe_2_O_3_@GMA@IM are shown in Figure 3. The stretching vibration peak of Fe-Ois 578 cm^−1^. The −CH=CH_2_ characteristic absorption peak at 1630 cm^−1^ of the synthesized monomer GMA disappeared after the preparation of the magnetic polymer, proving the successful progress of the polymerization reaction [21]. The absorption peak at 903 cm^−1^ originated from the epoxy group of the polymer, which disappeared after the grafting reaction. For γ-Fe_2_O_3_@GMA@IM, its absorption peaks at 940 cm^−1^ and 1169 cm^−1^ are C-N stretching vibrations of imidazole groups and C=N stretching vibrations at 1570 cm^−1^ [22]. In addition, γ-Fe_2_O_3_@GMA@IM forms a strong −OH characteristic absorption peak at 3400 cm^−1^, which also confirms that the epoxy groups of the polymer γ-Fe_2_O_3_@GMA are opened during the grafting process, and -OH is formed [23].

**Figure 4:**
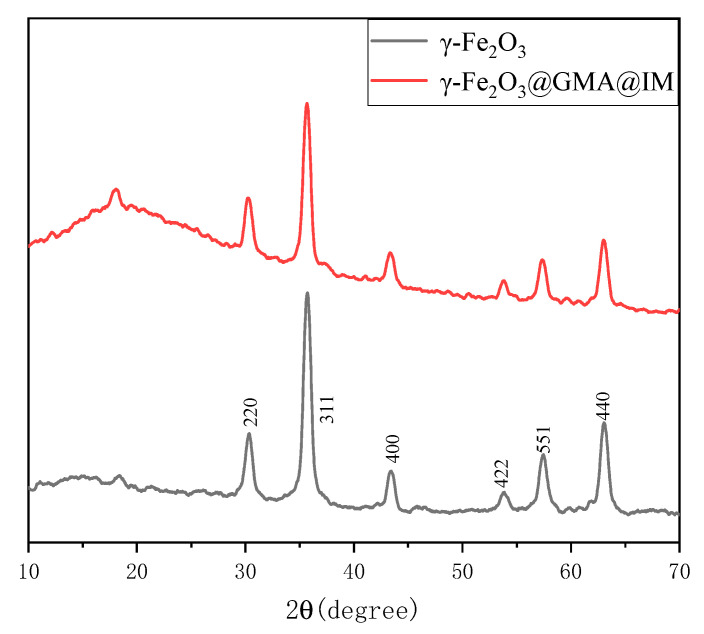
The XRD pattern of γ-Fe_2_O_3_@GMA@IM.

According to the XRD patterns of γ-Fe_2_O_3_@GMA@IM and γ-Fe_2_O_3_ shown in Figure 4, the magnetic polymer has a broad peak in the range of 10°–25° at 2θ, which is the characteristic peak of the amorphous material, which should be the polymer group generated. In contrast to standard PDF cards (39-1346), absorption peaks corresponding to (220), (311), (400), (422), (551) and (440) crystal faces at 2θ = 30.36°, 35.72°, 43.40°, 53.84°, 57.48°, 63.02°, further confirming that the crystal form of γ-Fe_2_O_3_ did not change with the reaction.

**Figure 5:**
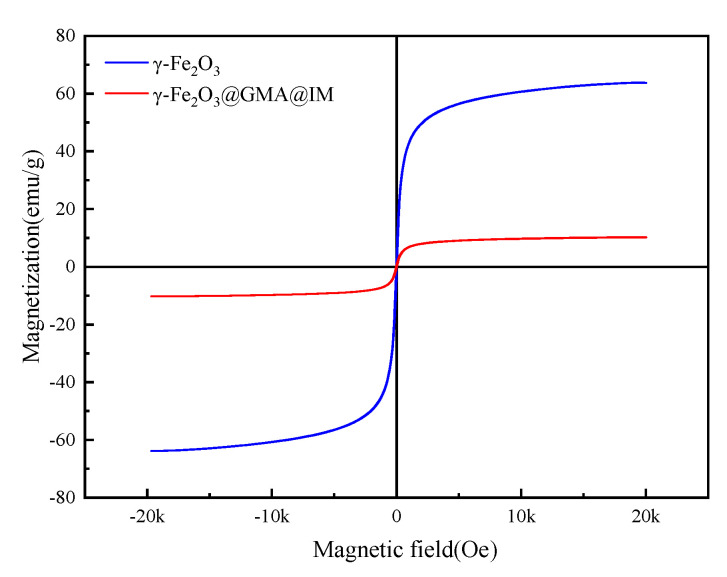
The hysteresis loop of γ-Fe_2_O_3_@GMA@IM.

**Figure 6:**
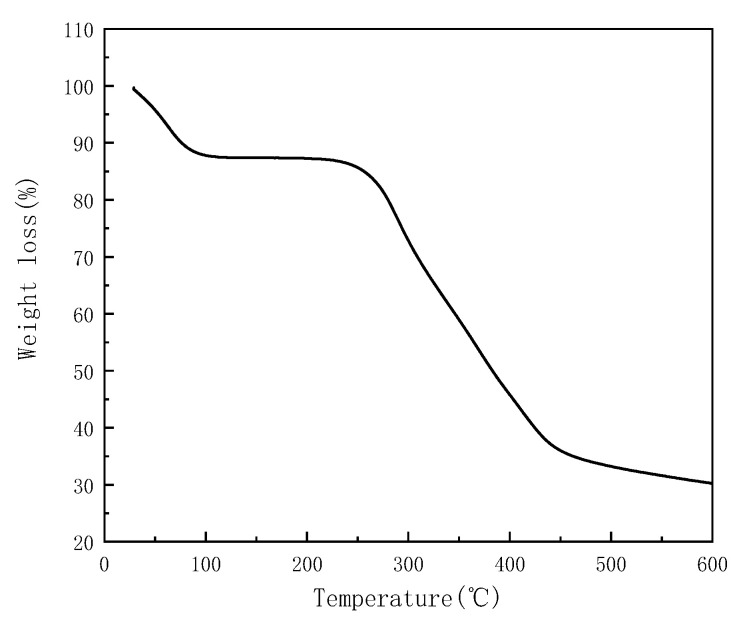
Thermogravimetric curve of γ-Fe_2_O_3_@GMA@IM.

The hysteresis loops of γ-Fe_2_O_3_ and γ-Fe_2_O_3_@GMA@IM are shown in Figure 5. The specific saturation magnetization of γ-Fe_2_O_3_ is 63.725 emu/g, and the specific saturation magnetization of γ-Fe_2_O_3_@GMA@IM is 10.211 emu/g. This is due to the reduction in the specific saturation magnetization of the magnetic polymer caused by the organic matter wrapped around the γ-Fe_2_O_3_. On the other hand, because the magnetic polymers have a relatively high remanence and coercivity, and thus a permanent magnetism, they can attract each other, agglomerate and rapidly settle in the absence of an external magnetic field, and the separation effect is good. Figure 6 shows the thermal stability of γ-Fe_2_O_3_@GMA@IM. The weight loss of the substance below 100 °C is mainly due to the volatilization of the water vapor of the polymer; the thermal stability is good at 100–200 °C, indicating that the functional group of the substance is relatively stable. The main weight loss region of the ionic liquid-modified magnetic polymer is 250–450 °C, at which temperature, the polymer undergoes decarboxylation and carbonization reactions. The final magnetic polymer decomposes into inorganic residues.

### 3.2. Comparative Experimental Analysis

**Figure 7:**
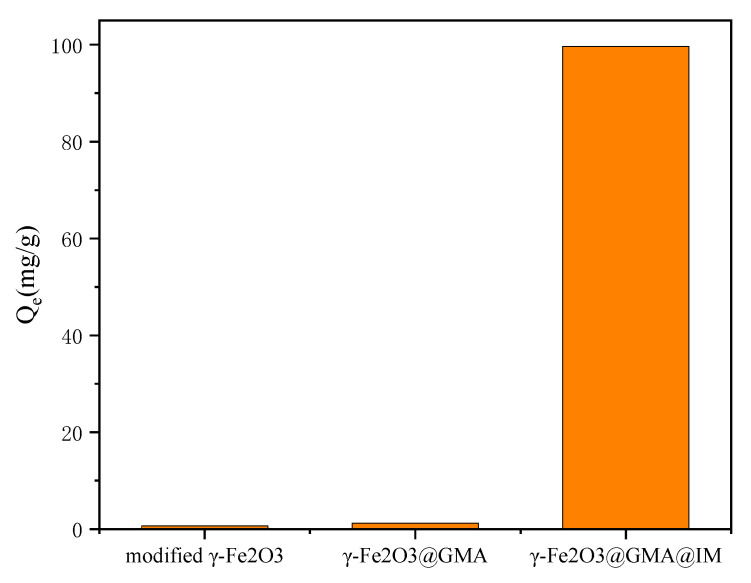
The adsorption capacity of different materials for FCF.

Figure 7 shows the maximum adsorption capacity of modified γ-Fe_2_O_3_, γ-Fe_2_O_3_@GMA and γ-Fe_2_O_3_@GMA@IM for FCF at an initial concentration of 100 mg/L. It can be seen that the adsorption capacity of modified γ-Fe_2_O_3_ and γ-Fe_2_O_3_@GMA on FCF is very small and can be ignored. At the same time, it is shown that the direct adsorption of FCF by modified γ-Fe_2_O_3_ and γ-Fe_2_O_3_@GMA can be ignored. Therefore, the main adsorption effect of γ-Fe_2_O_3_@GMA@IM on FCF comes from the grafted imidazole group.

### 3.3. The Influence of pH

**Figure 8:**
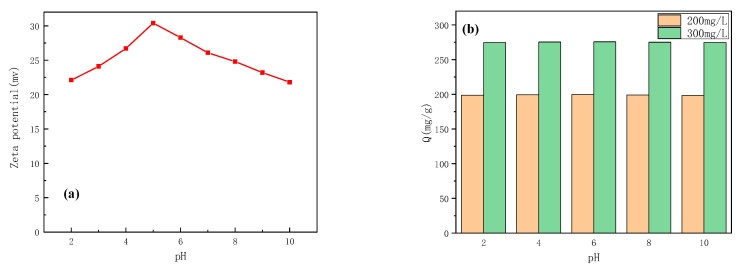
The zeta potential (**a**) and adsorption capacity (**b**) of γ-Fe_2_O_3_@GMA@IM at different pHs.

It is known that the initial pH of the solution can affect the removal rate by adjusting the zeta potential of the adsorbent and the ionization degree of the dye solution [24]. Therefore, it is of great significance to study the effect of pH on the adsorption of anionic dyes. In this study, 0.050 g γ-Fe_2_O_3_@GMA @IM was added into 50 mL ultra-pure water using Zetasizer Nano ZS90 Nanoparticle Size and Zeta Potential Analyzer (MALVERN, UK). The pH of the suspension was adjusted to 2–10 using HCl solution and NaOH solution, respectively. The zeta potential of the sample was measured. Figure 8a shows the zeta potential of the ionic liquid-modified magnetic polymer at pH 2–10. Additionally, at pH 5, its zeta potential is the highest, reaching 30.4 mV, which may be caused by imidazoline-like basic groups, such as amino and imino. Of course, we need to consider the specific data and discuss it. It can be seen from the figure that the polymer is positively charged in the pH range of 2–10. Figure 8b shows different pH values. Dye removal at two initial concentrations. It can be seen that the removal efficiency of FCF dye hardly changes at pH 2–10. The results show that the polymer can adsorb FCF dye in a wide range of pH. Further combining the data in Figure 7 and Figure 8a, it is shown that magnetic polymer adsorbs FCF mainly through electrostatic interaction between protic N on imidazole group and FCF.

### 3.4. Adsorption Kinetics

**Figure 9:**
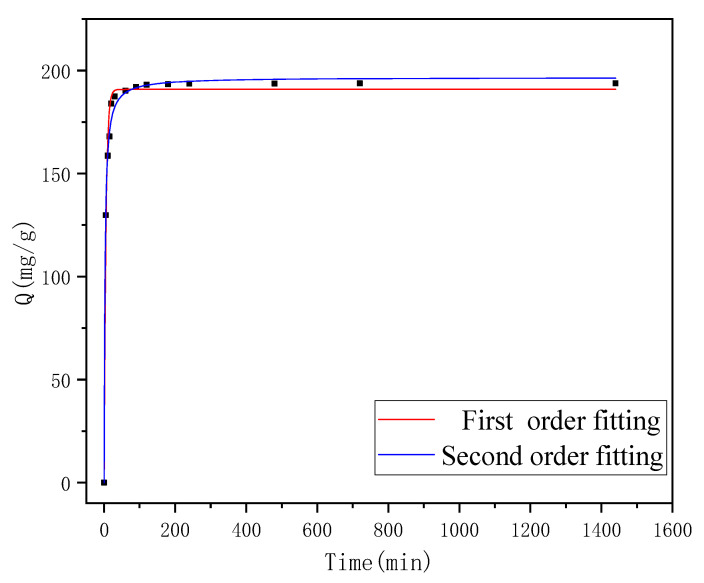
The adsorption kinetics of FCF on γ-Fe_2_O_3_@GMA@IM.

In order to investigate the variation in the adsorption amount of the ionic liquid-modified magnetic polymer on FCF with time during the adsorption process, the kinetic behavior of the polymer on FCF was investigated, and the results are shown in Figure 9. It can be seen that the initial adsorption rate of the polymer to FCF is fast, the adsorption amount increases rapidly, and then the adsorption gradually slows down, and finally reaches an equilibrium state. This process is related to the gradual saturation of the adsorption sites of the polymer. In order to further illustrate the adsorption kinetics of γ-Fe_2_O_3_@GMA@IM on FCF, the pseudo-first-order kinetics and pseudo-second-order kinetic models were used to fit the adsorption kinetic data. The equations are as follows:

Pseudo-first order kinetic equation: Q_t_ = Q_e_(1 − exp^−k^_1_^t^) (3)

Pseudo-second-order kinetic equation: Q_t_ = (k_2_tQ_e_^2^)/(1 + k_2_tQ_e_) (4)

In the formulae, Q_e_ (mg/g) is the equilibrium adsorption capacity, Q_t_ is the adsorption capacity at time, and k_1_ and k_2_ are pseudo-first-order and pseudo-second-order rate constants, respectively.

The adsorption kinetic simulation parameters are shown in Table 2. It can be seen from the data in the table that the adsorption behavior of the polymer to FCF is more in line with the pseudo-second-order kinetic equation (R^2^ > 0.99). The results indicate that the FCF molecule from the solution to magnetic polymer surface is controlled by a chemisorption step. Consistent with the previous corollary, “magnetic polymer adsorbs FCF mainly through electrostatic interaction between protic N on imidazole group and FCF”. The adsorption of magnetic polymer to FCF almost reached the maximum adsorption effect within 120 min. The equilibrium adsorption capacity fitted by the model is essentially consistent with the experimental results.

### 3.5. Adsorption Isotherm

**Figure 10:**
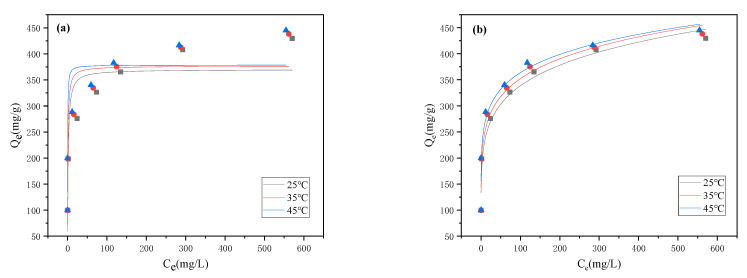
Langmuir adsorption isotherm (**a**) and Freundlich adsorption isotherm (**b**) of γ-Fe_2_O_3_@GMA@IM.

In order to investigate the distribution of FCF between the aqueous solution and ionic liquid-modified magnetic polymer at a specific temperature during the adsorption process, the effect of the adsorption isotherms of a magnetic polymer on FCF at three temperatures was studied in this paper. The results are shown in Figure 10. The adsorption isotherm data in this study were fitted by the Langmuir and Freundlich isotherm models. The Langmuir model assumes that the surface of the adsorbent is uniform, the adsorption performance is the same everywhere, and the adsorption process is monolayer adsorption. After a certain adsorption site is occupied, no further adsorption will occur on this site. The Freundlich model is an empirical formula used to describe the adsorption on heterogeneous surfaces. The Langmuir and Freundlich model formulas are:

Langmuir isotherm: Q_e_ = Q_m_K_L_C_e_/(1 + K_L_C_e_) (5)

Freundlich isotherm: Q_e_ = K_F_C_e_^1/n^ (6)

In the formula, Q_e_ (mg/g) and C_e_ are the equilibrium adsorption capacity and equilibrium concentration of the adsorbent in the equilibrium state, respectively. Q_m_ (mg/g) is the maximum theoretical adsorption capacity. K_L_ and K_F_ are equilibrium constants related to adsorption capacity in Langmuir and Freundlich models, respectively, and the constants are related to adsorption performance; n characterizes the binding strength of the Freundlich model adsorbent–adsorbate interaction.

It can be seen from the correlation coefficients in the Table 3 that the adsorption isotherms of magnetic polymers to FCF are more in line with the Freundlich equation, indicating that the adsorption of γ-Fe_2_O_3_@GMA@IM to FCF exhibits a multi-layer adsorption [25]; therefore, the adsorption process may have different adsorption effects, such as hydrophobic, π–π interactions and cation–π interactions, etc. From the simulated parameters, the n value of γ-Fe_2_O_3_@GMA@IM for FCF adsorption is greater than 1, which is a favorable adsorption, and the n value increases with the increase in temperature, which is consistent with the change trend of K_L_ and K_F_ [26,27]. However, the maximum adsorption amount does not change much with the increase in temperature, which may be because chemisorption is less affected by temperature.

### 3.6. Repeatability

The reusability of the adsorbent is one of the most important parameters for evaluating the quality of the adsorbent, and it is also a key indicator to determine whether it can be practically applied. In order to investigate the reusability of γ-Fe_2_O_3_@GMA@IM, 10% NaCl solution was used to regenerate the adsorption equilibrium polymer, and five batches of adsorption–regeneration experiments were continuously carried out.

**Figure 11:**
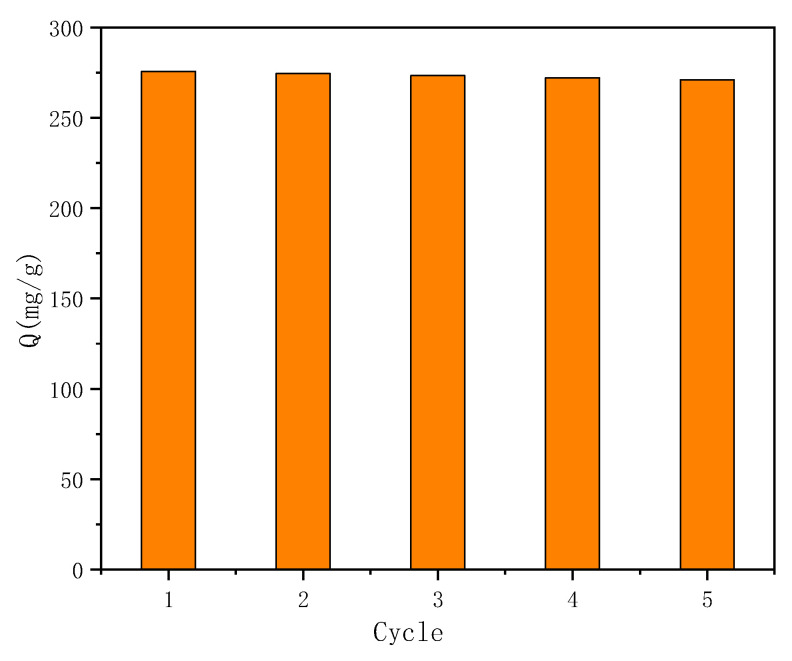
Reusability of γ-Fe_2_O_3_@GMA@IM adsorption FCF.

Figure 11 shows the experimental results of the adsorption–regeneration cycle stability of γ-Fe_2_O_3_@GMA@IM on FCF dyes. It was found that the maximum adsorption effect of the magnetic polymer on FCF was still 98.3% after repeated the experiment was repeated five times, indicating that the magnetic polymer had an excellent recycling performance for the adsorption of FCF.

## 4. Conclusions

In this study, the magnetic polymer γ-Fe_2_O_3_@GMA@IM was prepared by suspension polymerization and grafting. The magnetic polymer was used as an adsorbent to remove the anionic dye FCF from an aqueous solution. The properties of magnetic polymers were determined based on different characterization techniques. The adsorption effect of the polymer on FCF had no clear change between pH 2–10. By comparing with other data, it was shown that the magnetic polymer mainly adsorbed FCF through electrostatic interaction between protic N on imidazole group and FCF. By studying the adsorption kinetics, it was observed that the adsorption of FCF by the polymer could reach equilibrium within 120 min, which was more in line with the pseudo-second-order kinetic model. Compared with the Langmuir isotherm, the adsorption data were more in line with the Freundlich isotherm model, indicating that the adsorption of FCF by the polymer had some other effects besides ion exchange. By using 10% NaCl to desorb and regenerate the adsorbed polymer, after five adsorption-desorption processes, the adsorption effect could still reach 98.3%, showing a good regeneration performance. The polymer had a maximum adsorption capacity of 445 mg/g for FCF and was a good adsorbent.

## Figures and Tables

**Table 1:** Physicochemical parameters of γ-Fe_2_O_3_@GMA@IM.

Parameters	γ-Fe_2_O_3_@GMA@IM
Matrix structure	Acrylic
Water content (%)	55–60%
Diameter (μm)	50
Average pore size (nm)	3.421
BET specific surface area (m^2^/g)	14.703
Total pore volume (cm^3^/g)	0.042

**Table 2:** Adsorption kinetics simulation parameters.

Adsorbate	Pseudo-First-Order Model			Pseudo-Second-Order Model		
	k_1_	q_e_	R_1_^2^	k_2_	q_e_	R_2_^2^
FCF	0.1966	190.8	0.988	0.00215	196.7	0.996

**Table 3:** Adsorption isotherm parameters at different temperatures.

Adsorbate	T (°C)	Langmuir Model			Freundlich Model		
		K_L_	Q_max_	R^2^	n	K_F_	R^2^
FCF	25 °C	0.661	369.9	0.809	6.27	162.45	0.971
	35 °C	1.146	376.4	0.830	6.76	178.23	0.960
	45 °C	3.257	378.6	0.783	7.52	197.20	0.934

## Data Availability

Raw data is available upon request.
